# Nicotinic Agonist Inhibits Cardiomyocyte Apoptosis in CVB3-Induced Myocarditis via *α*3*β*4-nAChR/PI3K/Akt-Dependent Survivin Upregulation

**DOI:** 10.1155/2019/9496419

**Published:** 2019-03-07

**Authors:** Ping Li, Yaoyao Yan, Youyang Shi, Bo Cheng, Yi Zhan, Qiaoyu Wang, Qiaofang Ye, Yawen Weng, Tingting Wu, Rongzhou Wu

**Affiliations:** Children's Heart Center, The Second Affiliated Hospital and Yuying Children's Hospital of Wenzhou Medical University, Institute of Cardiovascular Development and Translational Medicine, The Second School of Medicine, Wenzhou Medical University, Wenzhou, Zhejiang 325027, China

## Abstract

**Background:**

Cardiomyocyte apoptosis is critical for the development of coxsackievirus B3- (CVB3-) induced myocarditis, which is a common cardiac disease that may result in heart failure or even sudden death. Previous studies have associated CVB3-induced apoptosis with the downregulation of antiapoptotic proteins. Here, attempts were made to examine whether nicotinic acetylcholine receptors (nAChRs), especially *α*3*β*4-nAChRs, were a novel therapeutic antiapoptotic target via the activation of survivin, a strong antiapoptotic protein, in viral myocarditis (VMC).

**Methods and Results:**

In the present study, we demonstrated that nAChRs, *α*3*β*4-nAChR subunits in particular, were present and upregulated in CVB3-infected neonatal rat cardiomyocytes (NRC) and H9c2 cells by RT-qPCR. The function of *α*3*β*4-nAChRs was next examined using its specific blocker *α*-CTX AuIB in vitro. The results of the TUNEL assay and western blot experiments showed that the block of *α*3*β*4-nAChRs abrogated nicotine-mediated protection of NRC from CVB3-induced apoptosis, and this effect displayed a substantial correlation with the protein expressions of pAkt, survivin, and Cleaved Caspase-3. Hence, the involvement of the PI3K/Akt pathway was further verified by LY294002, a selective inhibitor of PI3K. As a result, nicotine-mediated induction of pAkt and survivin was abolished by LY294002; meanwhile, apoptotic NRC were increased accompanied by an increase of Cleaved Caspase-3 expression. Regarding CVB3-infected BALB/c mice, the *α*-CTX AuIB- and LY294002-treated groups had a lower survival rate, deteriorative ventricular systolic function, and more severe inflammation than the nicotine-treated group and the modulation of pAkt, survivin, and Cleaved Caspase-3 protein expressions was similar to that in CVB3-infected NRC. In addition, we found that a nicotinic agonist reduced CVB3 replication in a dose-dependent manner in vitro, which indicates that nAChR activation may serve as a possible protection mechanism of CVB3-induced myocarditis.

**Conclusions:**

Our study demonstrated that *α*3*β*4-nAChR subunits are essential in the nicotine-mediated antiapoptotic effect of protecting cardiomyocytes from CVB3-induced apoptosis in vivo and in vitro. This protection correlated with the PI3K/Akt pathway and the inducement of the antiapoptotic protein survivin. A combination of these mechanisms serves as a novel protective response to treat viral myocarditis.

## 1. Introduction

Viral myocarditis (VMC) is a cardiac disease associated with the infiltration of inflammatory cells occurring during infection with a variety of viruses [1]. Although previous studies have recognized that chronic dilated cardiomyopathy, congestive heart failure, or even sudden unexpected death can be caused by VMC, a challenge still exists to diagnose and treat this difficult disease [2]. Coxsackieviruses, members of the Picornaviridae family, enterovirus genus, are the commonest cause of human myocarditis and have been widely applied to establish a murine model of myocarditis [3]. Hence, without exception, we focused on coxsackievirus B3- (CVB3-) induced myocarditis in our study. Although there is as yet no consensus about the specific pathogenesis of VMC, Kawano et al. [4] reported for the first time in 1994 that there was multifocal cardiomyocyte apoptosis in patients with chronic myocarditis by myocardial biopsy. Later on, Henke et al. [5] and Kyto et al. [6] observed a great number of apoptotic cardiomyocytes and a significant increase of Caspase-3 activity in mice infected with CVB3, indicating that the apoptosis of cardiomyocytes is involved in the pathogenesis of myocarditis. Thus, it would be essential to identify the mechanisms of cardiomyocyte apoptosis induced by CVB3 and attempts should be made to attenuate CVB3-mediated apoptosis.

**Figure 1:**
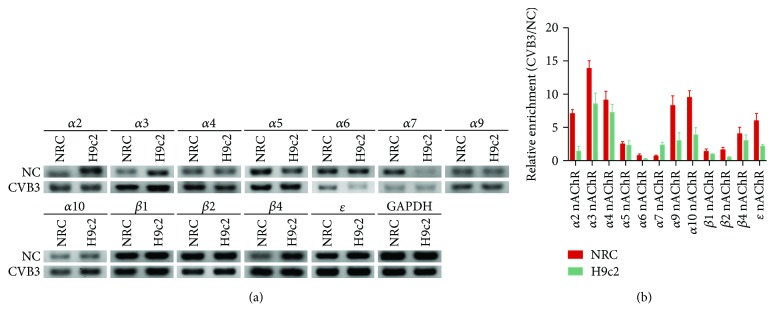
The mRNA expression of nAChRs in CVB3-infected NRC and H9c2 cells. NRC and H9c2 cells were cultured for 48 h in 10% FBS medium and then stimulated by CVB3 for 2 h. A continuous culture for 24 h was accepted after CVB3 was removed and total mRNA was then collected. PCR for GAPDH was regarded as the loading control. (a) RT-PCR showing the mRNA expression of nAChR subunits in NRC and H9c2 cells with or without CVB3 infection. (b) RT-qPCR showing the fold change of the mRNA expression of nAChR subunits in CVB3-infected NRC and H9c2 cells compared to normal controls.

Nicotinic acetylcholine receptors (nAChRs), comprising of 10 *α* (*α*1 to *α*10), 4 *β* (*β*1 to *β*4), *γ*, *δ*, and *ε* subunits symmetrically around a central pore [7], are ligand-gated ion channels [8] with multiple functions in muscle contraction [9], promoting angiogenesis, inhibiting inflammation, and in addition, protecting cells from apoptosis [10–12]. Previous studies have reported that nAChRs are expressed in rat heart with diverse functions [9, 13, 14]. We therefore examined the mRNA expression of nAChRs in neonatal rat cardiomyocytes (NRC) and H9c2 cells (a cell line derived from embryonic BD1X rat heart tissue), and we found a higher expression of nAChRs after these cells were infected with CVB3 for 24 h, especially *α*3- and *β*4-subunits (Figure 1). We speculated that the activation of nAChRs could attenuate apoptosis after CVB3 infection and *α*3*β*4-nAChR are the dominant functional subunits. A series of experiments were designed to examine this hypothesis.

**Figure 2:**
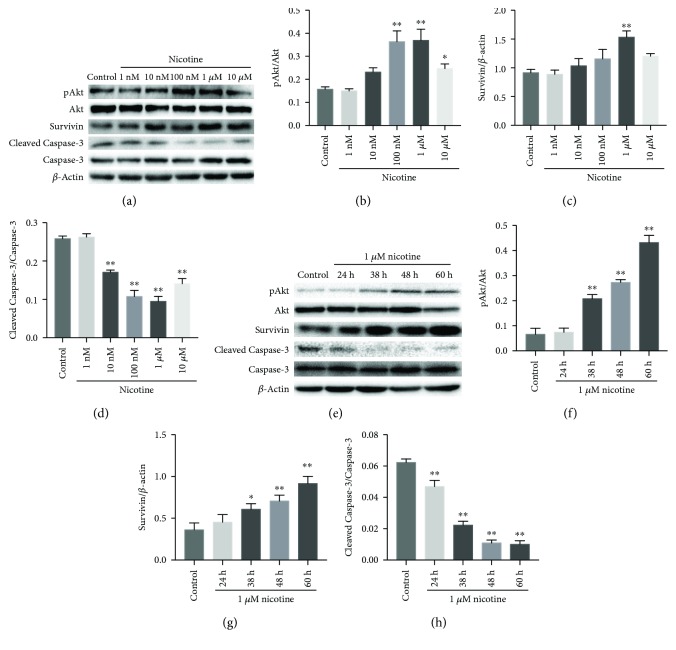
The involvement of survivin in the antiapoptotic effect of a nicotinic agonist. (a-d) NRC were exposed to CVB3 for 2 h and then treated with nicotine (0, 0.001, 0.01, 0.1, 1, and 10 *μ*M) for 36 h. The total proteins were then collected for western blot. ^∗∗^*P* < 0.01 vs. control group. (e-h) NRC were exposed to CVB3 for 2 h and then treated with 1 *μ*M nicotine for different times (24 h, 36 h, 48 h, and 60 h). The control group was cultured for 24 h in the absence of nicotine after CVB3 infection. The total proteins were then collected for western blot. ^∗∗^*P* < 0.01 vs. control group.

The antiapoptotic action of nicotine, a nAChR agonist, has been recently attributed to its function in activating antiapoptotic proteins like Bcl-2, XIAP, and survivin, phosphorylating Akt, and deregulating proapoptotic proteins like Bax and Bad [15–17]. Here, we discovered a time-dependent manner of survivin expression in CVB3-infected NRC (Figure 2(e)); therefore, we included a set of experiments to clarify whether nicotine attenuates cardiomyocyte apoptosis induced by CVB3 by upregulating survivin and whether PI3k/Akt pathway is involved in this effect.

## 2. Materials and Methods

### 2.1. Murine Viral Myocarditis and Groups

Four-week-old male BALB/c mice, purchased from the Shanghai Laboratory Animal Center (SLAC), China, were inbred under a specific pathogen-free environment at the Animal Experiment Center of Wenzhou Medical University. All experiments were performed with the approval of the Wenzhou Medical University Ethics Committee and in accordance with the China Animal Welfare Legislation, as well as the guide for the care and use of laboratory animals. The mice were randomly divided into five groups: the normal control group (NC), the viral myocarditis group (VMC), the nicotine (Sigma-Aldrich, N3876, 0.2 mg/kg/d, i.p.) treatment group, the nicotine and *α*-Conotoxin AuIB [18] (Tocris Bioscience, 700 pmol/mouse/d, i.p.) treatment group, and the nicotine and LY294002 (Selleck Chemicals, 0.4 mg/kg/d, i.p.) treatment group. 1.0 × 10^6^ plaque-forming units of purified CVB3 (Nancy strain) in 0.1 ml phosphate-buffered saline (PBS) were i.p. injected in the mice from the VMC group and the treatment groups on the 1st day of the experiment. Normal controls received PBS instead of CVB3. Animals of each group (*n* ≥ 8) were sacrificed at day 7 postinfection, and hearts were snap-frozen and stored at −80°C for analysis.

### 2.2. Cell Culture and Infection

0- to 3-day-old neonatal SD rat pups were purchased from SLAC and sacrificed for the extraction of cardiomyocytes as previously described [19]. H9c2 cells, purchased from the American Type Culture Collection (ATCC, Manassas, VA, USA), were cultured in high-glucose DMEM (Gibco) with 10% fetal bovine serum (FBS) with 1% penicillin/streptomycin in a stable environment of 5% CO_2_ and 21% O_2_ with the temperature maintained at 37°C. Neonatal rat cardiomyocytes (NRC) and H9c2 were exposed to CVB3 at a multiplication of infection (MOI) of 5 and 15, respectively, for 2 h under serum starvation conditions to establish a cell model of viral myocarditis. Control groups were treated with serum-free medium for 2 h. After that, NRC and H9c2 cells were cultured in DMEM/F12 or high-glucose DMEM, respectively, with 10% FBS and stimulated with 1 *μ*M nicotine in the absence or presence of the indicated inhibitors for 36 h.

### 2.3. Cell Viability

NRC were planked into 96-well plates at a concentration of 8 × 10^3^ cells per well and were cultured with 10% FBS for 24-48 h. Thereafter, unattached cells were washed out with PBS and NRC were exposed to a freshly prepared nicotine and LY294002 solution in 2% FBS at the required concentration for 24 h. Later, 5 mg/ml MTT (Sigma-Aldrich) was added to each well and then incubated for another 4 h. 100 *μ*l DMSO was added to resolve the purple formazan crystals after MTT and culture medium were removed. The number of viable cells was measured by evaluating the absorbance at 570 nm.

### 2.4. Q-PCR and Northern Blotting

Total RNA was isolated from NRC and H9c2 cells by TRIzol Reagent (Invitrogen) and reversely transcribed to synthesize cDNA (ReverTra Ace qPCR RT Kit, TOYOBO) according to the manufacturer's protocol. The expressions of *α*2-, *α*3-, *α*4-, *α*5-, *α*6-, *α*7-, *α*9-, *α*10-, *β*1-, *β*2-, *β*4-, *ε*-nAChR, CVB3, and GAPDH mRNA were detected via the SYBR Green real-time fluorescence quantitative method (SYBR® Green Realtime PCR Master Mix, TOYOBO). All of the mRNA primer pairs (shown in Table 1) were synthesized in Sangon Biotech Co., Ltd. (Shanghai, China). Additionally, mRNA expressions of the subtypes of nAChRs on NRC and H9c2 cells after 24 h CVB3 infection were determined by using relative quantification and the ratios shown below were compared to normal controls. The CVB3 copy number of NRC and H9c2 cells which were treated with varying levels of nicotine for 36 h was also detected and the results were compared to CVB3-infected controls. 10 *μ*g of total RNA was used for northern blotting. PCR analysis was repeated three times for consistency.

### 2.5. Echocardiography

7 days after CVB3 inoculation, transthoracic echocardiography was performed by using the Vevo 1100 Imaging System (VisualSonics, CA) with a 30 MHz high-frequency transducer. The mouse was positioned on a heating pad to maintain body temperature at 37°C and anesthetized by inhaling isoflurane. The echocardiographic ventricular structure was visualized by a blinded investigator in the left parasternal long-axis view, and the left systolic function (LVEF) was measured as previously described [20]. Other parameters, including the left ventricular (LV) posterior wall end-diastolic and end-systolic thickness, interventricular septal end-diastolic and end-systolic thickness, LV end-diastolic and end-systolic diameter, LV end-diastolic and end-systolic volume, and LV fractional shortening were also measured. For the VMC group and the treatment groups, the heart rate was around 230 beats per minute. All the echocardiographic data were collected and averaged over three consecutive cardiac cycles.

### 2.6. Western Blot Analysis

Total protein was extracted from homogenized NRC and myocardial tissue samples, and phosphatase inhibitor (PhosSTOP, Roche) was added on the basis of the manufacturer's instructions. The protein concentration was measured by BCA assay (Pierce™ BCA Protein Assay Kit, Thermo Fisher Scientific). The proteins were separated by 12% SDS-PAGE and wetly transferred to 0.22 *μ*m PVDF membrane (Millipore). After 2h of continued blocking by 5% skim milk dissolved in TBS, the membranes were, respectively, incubated in monoclonal antibodies, including pAkt (1 : 1000, Ser473, #4060, Rabbit mAb, CST), Akt (1 : 1000, #4691, Rabbit mAb, CST), survivin (1 : 1000, #2808, Rabbit mAb, CST), Cleaved Caspse-3 (1 : 1000, #9661, Rabbit mAb, CST), Caspase-3 (1 : 1000, #9665, Rabbit mAb, CST), and *β*-actin (1 : 1000, ab8227, Rabbit mAb), at 4°C overnight. The membranes were then washed in TBST (0.1% Tween 20 in TBS) and incubated with goat anti-rabbit IgG (CST) for 2 h at room temperature (RT). The ECL developer (Thermo Fisher Scientific) was used, and the images were analyzed by the Bio-Rad Gel Imaging System, while the gray value was measured with Image Lab software.

### 2.7. Evaluation of Apoptosis by TUNEL Assay

Apoptosis was assessed by transferase-mediated deoxyuridine triphosphate-biotin nick-end labeling (TUNEL, In Situ Cell Death Detection Kit, Roche) assay. Adherent cells cultured on chamber slides after respective treatments were fixed with a freshly prepared 4% paraformaldehyde for 1 hr at RT and then blocked with 3% H_2_O_2_ for 10 min. 0.1% Triton X-100 was used for 10 min on ice to permeabilize the cells. The sample was then covered by 50 *μ*l TUNEL reaction mixture and incubated for 1 h at 37°C in a humidified and dark atmosphere. Samples were analyzed in a drop of antifade mounting medium (Thermo Fisher Scientific) under a fluorescence microscope, and the fields of view were randomly selected.

### 2.8. Histopathology

Formalin-fixed and paraffin-embedded hearts from mice in each group were sliced up into 5-*μ*m-thick tissue sections and stained with hematoxylin and eosin (Hematoxylin-Eosin (HE) Staining Kit, Solarbio). To evaluate inflammation and myocardial injury, a minimum of 5 replicates of each sample were required for the analysis of each group, and 5 visual fields were measured in each replicate. The severity of myocarditis was assessed by the percentage of the infiltrating mononuclear cells and myocyte necrosis as described previously [21].

### 2.9. Statistical Analysis

Data were presented as mean ± standard error of the mean (SEM). Normality was determined using the Shapiro-Wilk test. As the differences of all parameters passed the normality test (*α* = 0.05) and there were no significant differences in variance between groups (*F*-test), one-way analysis of variance (ANOVA) followed by post hoc tests was used for the comparisons of multiple groups. GraphPad Prism-7 statistic software (GraphPad Software Inc., La Jolla, CA) was adopted for data analysis. Values of *P* < 0.05 was considered statistically significant.

## 3. Results

### 3.1. The mRNA Expression of nAChRs in CVB3-Infected NRC and H9c2 Cells

Preliminary studies revealed an antiapoptotic effect of nAChRs in human lymphocytes [22] and tumor cells [15, 23, 24]. Recent studies have also shown that *α*7-nAChR has anti-inflammation activities in autoimmune [25] and CVB3-induced myocarditis [26]. Thus, attempts were made to compare the mRNA expression of nAChR subunits in NRC and H9c2 cells in the presence or absence of CVB3 infection by using RT-PCR and RT-qPCR. As is shown in Figure 1, the mRNA expression of *α*2-, *α*3-, *α*4-, *α*5-, *α*9-, *α*10-, *β*4-, and *ε*-subunits were both upregulated in the two kinds of cells. Additionally, the mRNA expression of *α*3- and *β*4-subunits had the most obvious upregulation, respectively, regarding *α*-subunits and *β*-subunits. These results suggest that *α*3- and *β*4-subunits may play a vital role in CVB3-induced myocarditis.

### 3.2. Nicotinic Agonist Reduces CVB3 Replication in a Dose-Dependent Manner In Vitro

**Figure 3:**
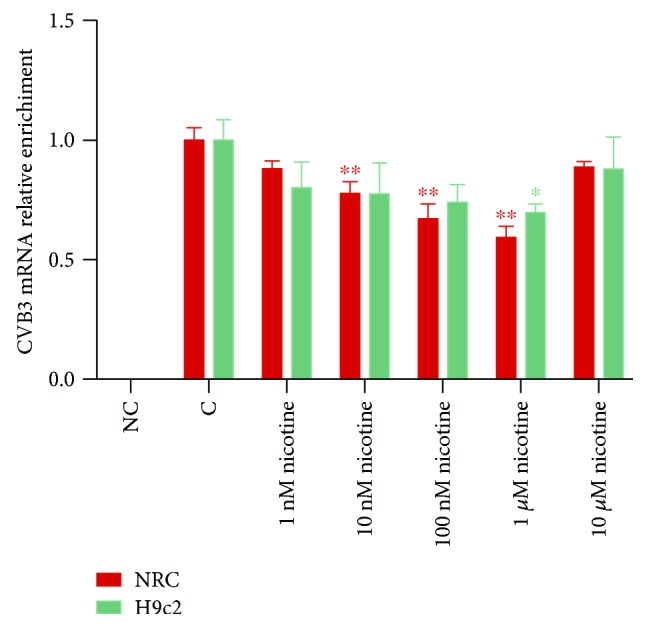
The mRNA expression of CVB3 in NRC and H9c2 cells after treatment with different concentrations of nicotine. After being infected by CVB3 for 2 h, the culture medium was removed and different concentrations of freshly dissolved nicotine were added to stimulate nAChRs. Total mRNA was collected 36 h later for the evaluation of CVB3 mRNA by RT-qPCR. ^∗^*P* < 0.05 and ^∗∗^*P* < 0.01 vs. the control group.

In acute and subacute stages of viral myocarditis, the massive replication, propagation, and spread of CVB3 will make a direct contribution to the injury and necrosis of cardiac tissue. In order to estimate the impact of nAChR activation on CVB3 replication, a search for CVB3 mRNA expression by RT-qPCR was conducted and as exhibited in Figure 3, at a low concentration from 1 nM to 1 *μ*M, nicotine had a positive effect on attenuating CVB3 replication in NRC and H9c2 cells with the increase of concentration. However, at a higher dose of 10 *μ*M, this attenuation effect started rebounding.

### 3.3. Nicotinic Agonist Promotes Cell Viability at Low Concentrations and Protects NRC from CVB3-Induced Apoptosis in a Dose-Dependent Manner

**Figure 4:**
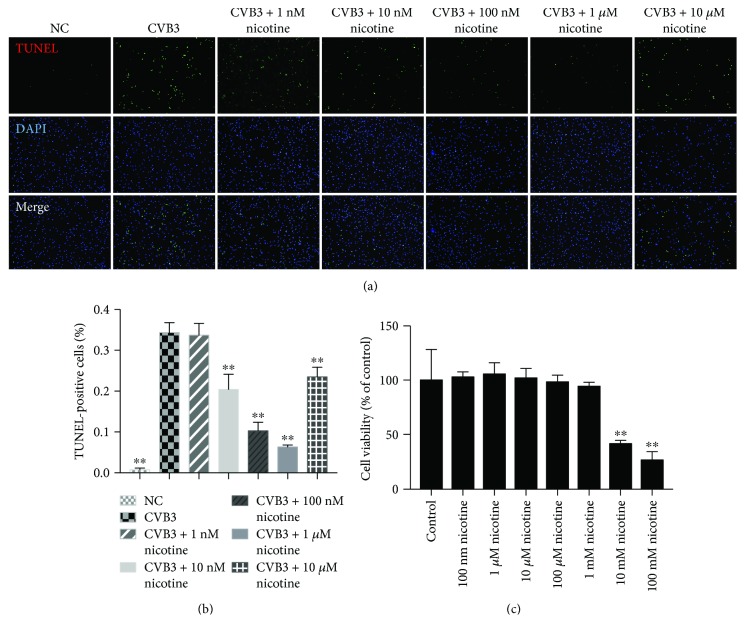
A nicotinic agonist promotes cell viability at low concentrations and protects NRC from CVB3-induced apoptosis in a dose-dependent manner. After being stimulated by CVB3 for 2 h, different concentrations of freshly dissolved nicotine were added to NRC for 36 h in 10% FBS medium before the TUNEL assay. The MTT assay was performed after treatment by nicotine (0, 0.1, 1, 10, 100, 1000, 10^4^, and 10^5^*μ*M) for 36 h, and no groups had CVB3 infection. (a) Photomicrographs (×100) of apoptotic cells. (b) Quantitative analysis of the number of TUNEL-positive cells. ^∗∗^*P* < 0.01 vs. the CVB3-infected group. (c) MTT assay assessing cell viability of NRC treatment with different concentrations of nicotine. ^∗∗^*P* < 0.01 vs. the control group.

Acting as an agonist at most nAChRs, nicotine was used to examine the antiapoptotic effects of nAChRs in CVB3-infected NRC. Measuring by TUNEL assay in Figures 4(a) and 4(b), we found that low concentrations of nicotine, including 10 nM, 100 nM, 1 *μ*M, and 10 *μ*M, could suppress apoptosis induced by CVB3 and 1 *μ*M nicotine had the superior effect. The MTT assay (Figure 4(c)) showed that the cell viability of the 1 *μ*M nicotine-treated group was improved compared to that of the normal control group.

### 3.4. The Involvement of Survivin in the Antiapoptotic Effect of the Nicotinic Agonist

Nicotine activates survivin protein expression in quite a few kinds of cells [23, 24, 27] by regulating the expression of phospho-Akt; thus, it plays a key role in the antiapoptotic process. On the basis of that information, the effect of nicotine on the levels of pAkt, survivin, and Cleaved Caspase-3 was examined by western blot. A similar upregulation of pAkt and survivin was found upon the treatment of NRC with nicotine in a time- and dose-dependent manner (Figure 2). When treated with 1 *μ*M nicotine, which had the superior effect of inhibiting apoptosis (Figure 4(a)), pAkt and survivin got the most obvious upregulation. Survivin and pAkt protein expressions were increased along with the prolonged treating time of 1 *μ*M nicotine (Figure 2(e)). Reversely, Cleaved Caspase-3 was downregulated when treated with nicotine compared to controls (Figure 2(d)). The comparison of short-time (24-36 h) and long-time (48-60 h) treatments with 1 *μ*M nicotine suggests a marked decrease on Cleaved Caspase-3 protein expression (Figure 2(h)). These results encouraged us to hypothesize that nicotine protects NRC from CVB3-induced apoptosis by upregulating pAkt and survivin protein expression, and caspase-3 also performs a vital role in this process.

### 3.5. *α*3*β*4-nACh-Related PI3K/AKT Signaling Pathway Involved in Antiapoptotic Effects of Nicotinic Agonist in CVB3-Induced Apoptosis of NRC

**Figure 5:**
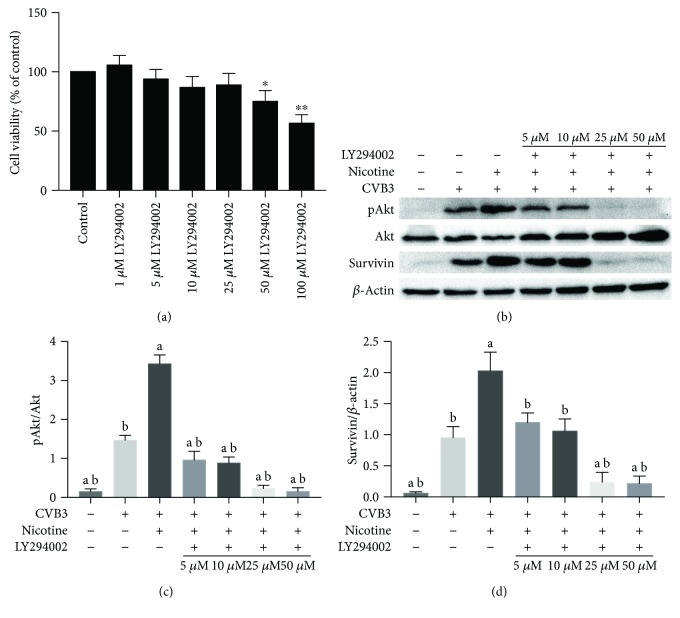
The PI3K/AKT signaling pathway involved in the antiapoptotic effects of a nicotinic agonist in the CVB3-induced apoptosis of NRC. (a) The MTT assay assessing the cell viability of NRC treated with different concentrations of LY294002. ^∗^*P* < 0.05 and ^∗∗^*P* < 0.01 vs. the control group. (b-d) After being stimulated by CVB3 for 2 h, LY294002 was used to ablate nicotine-induced inhibition of pAkt and survivin expression for 1 h. 1 *μ*M nicotine was then added to the culture for another 36 h. The expression of pAkt and survivin was measured by western blot. ^a^*P* < 0.05 vs. the CVB3-infected group. ^b^*P* < 0.05 vs. the nicotine-treated group.

**Figure 6:**
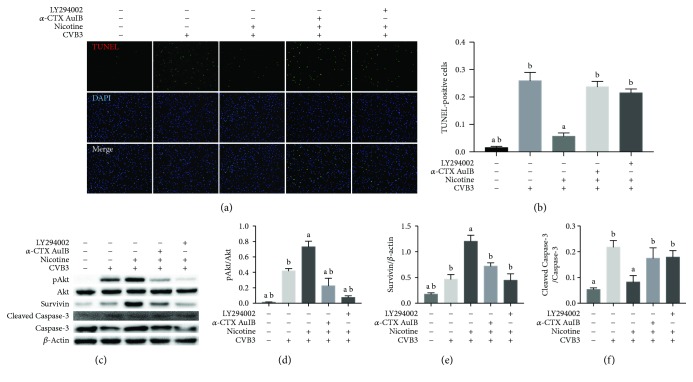
The *α*3*β*4-nAChR-related PI3K/AKT signaling pathway is involved in the antiapoptotic effects of a nicotinic agonist in the CVB3-induced apoptosis of NRC. Stimulated by CVB3 for 2 h, NRC were exposed to 25 *μ*M LY294002 and 5 *μ*M *α*-CTX AuIB for 1 h, respectively, to inhibit PI3K and *α*3*β*4-nAChR. 1 *μ*M nicotine was then used to activate nAChRs for 36 h. ^a^*P* < 0.05 vs. the CVB3-infected group. ^b^*P* < 0.05 vs. the nicotine-treated group. (a) Photomicrographs (×100) of apoptotic cells. (b) Quantitative analysis of the number of TUNEL-positive cells. (c-e) The expression of pAkt, survivin, and Cleaved Caspase-3 was measured by western blot.

The phosphatidylinositol 3-kinase (PI3K)/AKT pathway has been mentioned in studies of nicotine function [28, 29]; thus, attempts were made to clarify the role of this pathway in nicotine-mediated antiapoptotic effects on CVB3-infected NRC by using a specific blocker of PI3K (LY294002). As shown in Figure 5, the application of 25 *μ*M LY294002 abrogated nicotine-induced upregulation of pAkt and survivin, while limited impact on cell viability in NRC remained. An Akt-dependent characteristic was also detected in the regulation of the protein levels of survivin, as the protein expressions of pAkt and survivin both trended upwards under the treatment of nicotine and declined sharply under the application of 25 *μ*M LY294002. Furthermore, we examined whether nicotine ablated CVB3-induced apoptosis by upregulating survivin in an Akt-dependent manner. As displayed in Figures 6(a) and 6(b), 25 *μ*M LY294002 collapsed the nicotine-mediated protection against CVB3-induced apoptosis and Cleaved Caspase-3 was involved in this procedure (Figure 6(c)). We previously discovered an upregulation of *α*3*β*4 nAChR gene transcription in CVB3-infected NRC (Figure 1), which led us to use *α*-Conotoxin AuIB (*α*-CTX AuIB) as a selective blocker of *α*3/*β*4 nAChR to further ascertain the specific nAChR subunits involved in nicotine-mediated antiapoptotic effects on CVB3-infected NRC. As shown in Figures 6(a) and 6(b), *α*-CTX AuIB could ablate the nicotine-mediated antiapoptotic effect and the nicotinic upregulation of pAkt and survivin was abrogated by *α*-CTX AuIB (Figures 6(d) and 6(e)). Moreover, the protein expression of Cleaved Caspase-3 was increased, suggesting a caspase-dependent manner in the nicotine-mediated inhibition of apoptosis.

### 3.6. Nicotinic Agonist Attenuates Inflammation in the Murine Model of CVB3-Induced Myocarditis via *α*3*β*4-nAChR/PI3K/Akt-Dependent Pathway

The function of the nicotinic agonist was further examined by HE staining of the heart in the murine model of CVB3-induced myocarditis (Figure 6). Compared with normal controls, inflammatory infiltrates were serious after 7-day CVB3 infection, while nicotine treatment significantly attenuated inflammation. *α*-CTX AuIB and LY294002, on the contrary, reversed the therapeutic function of nicotine, and the inflammatory infiltrates in these two groups were even worse than those in the CVB3-infected group, indicating that nicotine has an anti-inflammatory action in the murine model of CVB3-induced myocarditis through the *α*3*β*4-nAChR/PI3K/Akt-dependent pathway.

### 3.7. Nicotinic Agonist Increases Survival Rate and Enhances Heart Function in the Murine Model of CVB3-Induced Myocarditis via *α*3*β*4-nAChR/PI3K/Akt-Dependent Survivin Upregulation

**Figure 7:**
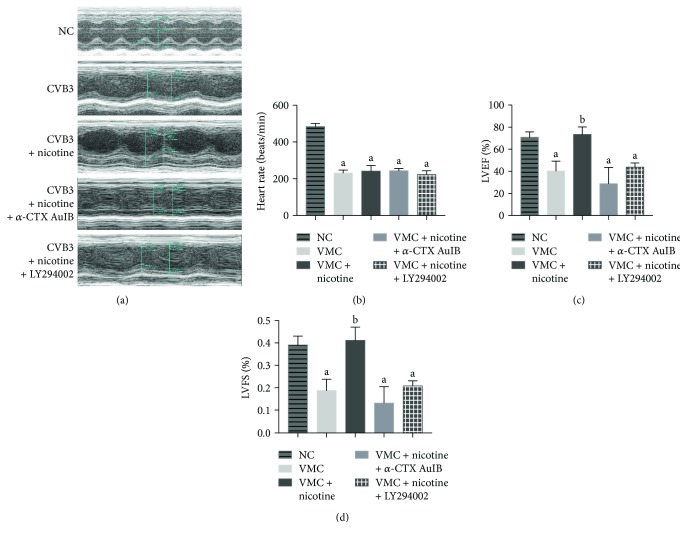
A nicotinic agonist enhances heart function in a murine model of CVB3-induced myocarditis via *α*3*β*4-nAChR/PI3K/Akt-dependent survivin upregulation. The M-type echocardiography was performed on day 7 after CVB3 inoculation (*n* = 8 per group). (a) A decreased heart rate was observed in CVB3-infected mice. (b) The nicotine-treated group reversed the severe reduction of contractile reserve caused by CVB3 infection, while *α*-CTX AuIB and LY294002, on the other hand, ablated this effect of nicotine, measured as changes in LVEF (c) and LVFS (d). ^a^*P* < 0.05 vs. the CVB3-infected group. ^b^*P* < 0.05 vs. the nicotine-treated group.

**Figure 8:**
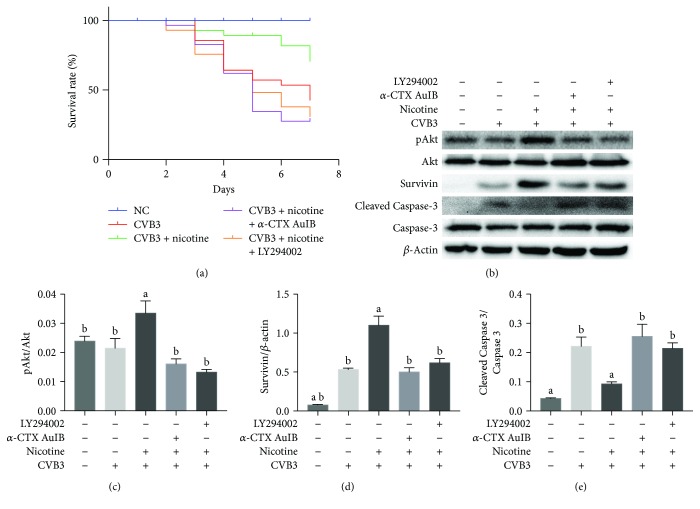
A nicotinic agonist enhances the survival rate in the murine model of CVB3-induced myocarditis via *α*3*β*4-nAChR/PI3K/Akt-dependent survivin upregulation. (a) Kaplan-Meier survival analysis of viral myocarditis mice in the study. Survival proportions at day 7 were 100% for the normal control group, 42.9% for the CVB3-infected group, 70.7% for the nicotine-treated group, 27.6% for the *α*-CTX AuIB-treated group, and 31.0% for the LY294002-treated group. (b) The expression of pAkt, survivin, and Cleaved Caspase-3 was measured by western blot. ^a^*P* < 0.05 vs. the CVB3-infected group. ^b^*P* < 0.05 vs. the nicotine-treated group.

It was next examined whether nAChR activation could protect cardiomyocytes in vivo from CVB3-induced apoptosis and whether the molecular mechanisms were underlying the induction of survivin. 7 days after CVB3 inoculation, echocardiography was adopted to measure the heart function. As shown in Figure 7, nicotine attenuated the reduction of contractile reserve and decrease of ventricular wall motion caused by CVB3. *α*-CTX AuIB and LY294002, however, suppressed this therapeutic action and caused a significant reduction in LVEF and LVFS (Figures 7(c) and 7(d)). The heart rate measured during echocardiography in CVB3-inoculated groups was similar at a level lower than normal controls due to the effect of CVB3 infection. Survival analysis (Figure 8(a)) of viral myocarditis mice in this study shows a consistent phenomenon with echocardiography. Up to the 7th day of experiments, there was significantly worse survival in the CVB3-infected group compared with normal controls. A decreased mortality was also observed in the nicotine-treated group. In *α*-CTX AuIB- and LY294002-treated groups, the survival rates were lower compared with the CVB3-infected group. The molecular mechanisms of nicotine on the expression of pAkt, survivin, and Cleaved Caspase-3 were examined by western blot. As shown in Figure 8(b), pAkt and survivin were upregulated under the treatment of nicotine yet downregulated by an *α*3*β*4-nAChR blocker (*α*-CTX AuIB) and a PI3K/Akt inhibitor (LY294002). The apoptosis related protein Cleaved Caspase-3 had an increased expression when treated with nicotine and a declined expression when exposed to *α*-CTX AuIB and LY294002, suggesting that nicotine has an antiapoptotic effect in the murine model of CVB3-induced myocarditis through *α*3*β*4-nAChR/PI3K/Akt-dependent survivin upregulation.

## 4. Discussions

Although the mechanism of viral myocarditis often remains unclear, it has been widely accepted that the inhibition of cellular apoptosis is essential in the treatment of viral myocarditis [30–32]. Early studies summarized that *α*7*β*2, *α*3*β*2, *α*3*β*4, and *α*4*β*2 nAChRs are expressed in the outer membrane of mitochondria, and they abolish the release of proapoptotic substances in an ion channel-independent manner [33]. As an attempt to understand the function of nAChRs in CVB3-induced myocarditis, we contrasted the mRNA expression of nAChRs in normal NRC and CVB3-infected NRC. Afterwards, we found that the mRNA expression of nAChRs was markedly increased in CVB3-infected NRC (Figure 1), especially *α*3*β*4 subunits, and upon nicotine stimulation, the levels of apoptosis in CVB3-infected NRC declined in a dose-dependent manner (Figure 4(a)). On this basis, we next examined whether *α*3*β*4-nAChRs, the ganglion-type nicotinic receptor, can be proposed as a therapeutic target for attenuating apoptosis in CVB3-induced myocarditis. We discovered that the inhibition of *α*3*β*4-nAChRs by *α*-CTX AuIB in CVB3-infected NRC (1) restrained the nicotine-mediated antiapoptotic effect, (2) suppressed the protein expression of survivin and phosphorylation of Akt, and (3) upregulated the protein expression of Cleaved Caspase-3 (Figure 6). All of these discoveries are in accordance with the role of *α*3*β*4-nAChRs in protecting cardiomyocytes from CVB3-induced apoptosis. This role is further supported by the proofs that the *α*-CTX AuIB-treated mice group has a remarkable exacerbation of cardiac function (Figure 7) and a declined survival rate (Figure 8(a)) compared to the nicotine-treated mice group. These effects are associated with the reduced expression of survivin and pAkt and increased expression of Cleaved Caspase-3 (Figure 8(b)).Therefore, this study firstly provides strong evidence in support of *α*3*β*4-nAChR as a vital receptor in attenuating cardiomyocyte apoptosis in CVB3-induced myocarditis.

**Figure 9:**
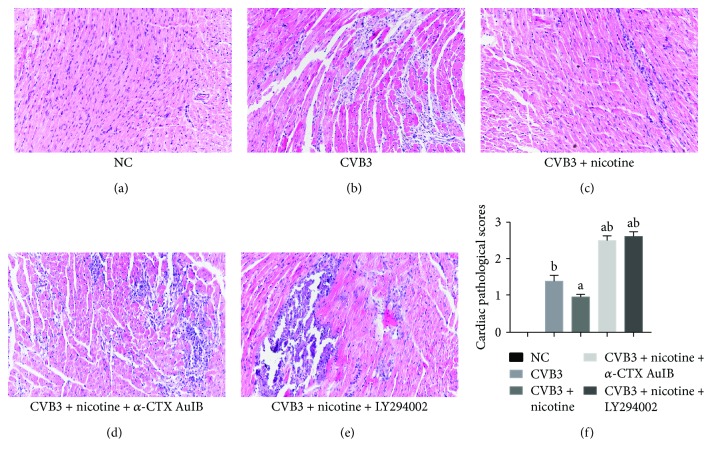
A nicotinic agonist attenuates inflammation in the murine model of CVB3-induced myocarditis via *α*3*β*4-nAChR/PI3K/Akt-dependent survivin upregulation. Mice from all groups were sacrificed on day 7 after CVB3 inoculation (*n* = 8 per group), and HE staining was performed thereafter. The scale bars are equal to 100 *μ*m in all of the images. (a-e) The histological findings of inflammation in heart tissue from different groups (magnification 200x), and there was no inflammation in the normal control group. (f) The cardiac pathological scores in different groups. ^a^*P* < 0.05 vs. the CVB3-infected group. ^b^*P* < 0.05 vs. the nicotine-treated group.

The role of Akt phosphorylation in mediating apoptosis has not been previously reported in CVB3-induced myocarditis, but it has been studied in multiple tumor models referring to the resistance against apoptosis induced by chemotherapeutic drugs [15, 34–36]. Quanri et al. [37] showed that nicotine stimulates the survival potential of human bronchial epithelial cells from deguelin-induced apoptosis via pAkt-related survivin upregulation. Our results also demonstrated that the nicotine-mediated antiapoptotic function dominantly relies on the PI3K/Akt pathway. After PI3K was inhibited by LY294002, a decrease of pAkt and survivin expression and an increase of TUNEL-positive cells and Cleaved Caspase-3 expression were observed in CVB3-infected NRC (Figure 9), while the expression trend of pAkt, survivin, and Cleaved Caspase-3 protein in the hearts of CVB3-infected mice was consistent with that in CVB3-infected NRC (Figure 8(b)). The upregulation of survivin by the PI3K/Akt pathway may be in very close contact with the stimulation of E2F1 and nuclear factor *κ*B (NF-*κ*B), which are important transcription factors involved in survivin expression [15, 38]. Alternatively, the PI3K/Akt pathway can stimulate E2F1 [39] and NF-κB [40] and then preferentially promote the expression of several genes involved in antiapoptotic pathways, for instance, survivin.

Survivin, also called baculoviral inhibitor of apoptosis repeat-containing 5 (BIRC5), belongs to the inhibitor of apoptosis (IAP) family [41] and acts as a key regulator of apoptosis in multiple cells, including stem cells [41] and tumor cells. The expression of survivin is abundant during embryonic and fetal development [42], but it remains at a low level during postnatal development. Interestingly, we found a time-related protein expression of survivin after CVB3 infection in NRC during nicotine treatment (Figure 2(e)). This finding suggests that survivin may play a vital role in CVB3-induced NRC apoptosis. In addition, Tamm et al. [43] previously proved that survivin binds to active caspase-3 and 7, the terminal effector cell death proteases, in 293 cells. Hence, we evaluated the involvement of the caspase pathway in the antiapoptotic mechanism of survivin in CVB3-infected NRC as well. The western blot experiment showed that the overexpression of survivin negatively correlates with the expression of Cleaved Caspase-3 in CVB3-infected NRC and the murine model of CVB3-induced myocarditis, indicating that the overexpression of survivin may protect NRC from CVB3-induced apoptosis by abolishing the activation of caspase-3.

In conclusion, our study has implicated for the first time the activation of *α*3*β*4-nAChRs by nicotine to the attenuation of CVB3-induced cardiomyocyte apoptosis in vivo and in vitro. In particular, this antiapoptotic effect mostly depends on the phosphorylation of Akt and upregulation of survivin. These results suggest a promising therapeutic target for reducing myocardial cell apoptosis in CVB3-induced myocarditis.

## Figures and Tables

**Table 1:** Primer sequences.

Gene	Forward primer	Reverse primer
GAPDH	5′-CAACTCCCTCAAGATTGTCAGCAA-3′	5′-GGCATGGACTGTGGTCATGA-3′
CVB3	5′-GTCGGGCTTTCATTTGCTTA-3′	5′-CTGGTTGGGCACTCCTGTAT-3′
*α*2-nAChR	5′-CACTACATTGCTGACCGTCTGAGG-3′	5′-ATGGAGGAAGGAAGAGTCCGATGG-3′
*α*3-nAChR	5′-GTCGCCTGCCGCTGTTCTAC-3′	5′-GTGGAAGGAATGGTCTCGGTGATC-3′
*α*4-nAChR	5′-CGTGTGGACCAACTGGACTTCTG-3′	5′-GCGTCGGATGATGAAGGCATAGG-3′
*α*5-nAChR	5′-CAACATCCACCACCGCTCTTCC-3′	5′-ATGCAATCGAGTGCGGCTTCC-3′
*α*6-nAChR	5′-GCTCTTCCACACGCTCTTCGC-3′	5′-GACGCAGCCACAGATTGGTCTC-3′
*α*7-nAChR	5′-CCTGGCTCTGCTGGTATTCTTGC-3′	5′-TCATGGTGCTGGCGAAGTATTGTG-3′
*α*9-nAChR	5′-CCTGGACAGCGGTGACCTCTC-3′	5′-TCGGAGCAGCAGCCATAGGAG-3′
*α*10-nAChR	5′-CAGCCTCCAGCCTCCACCAG-3′	5′-AGCGGTCCATTACTCTAGCCAGAC-3′
*β*1-nAChR	5′-GACCTGGAGTGGACCGACTACAG-3′	5′-CAGATTCAGCAGTGACACGGAGAG-3′
*β*2-nAChR	5′-GCGAAGTGAGGATGATGACCAGAG-3′	5′-TGGAGGAAGGTAGTGGCAGTGTAG-3′
*β*4-nAChR	5′-CCAGGACGGAGGACGGTGAAC-3′	5′-AGGATAGCCAGCGAGGTGATGAG-3′
*ε*-nAChR	5′-GGCTGTGCTGGAACTCGCTTAG-3′	5′-GCTTCTGCCTGTCTGCTTCTCAC-3′

## Data Availability

The data used to support the findings of this study are available from the corresponding author upon request.
